# ﻿First report of subfamily Scydmaeninae (Coleoptera, Staphylinidae) from Shanghai, with description of two new species

**DOI:** 10.3897/zookeys.1231.142538

**Published:** 2025-03-11

**Authors:** Zi-Wei Yin, Ting Feng, De-Yao Zhou

**Affiliations:** 1 Laboratory of Systematic Entomology, College of Life Sciences, Shanghai Normal University, Xuhui District, Shanghai 200234, China Shanghai Normal University Shanghai China; 2 Shanghai Zoo, 2381 Hongqiao Road, Changning District, Shanghai 200335, China Shanghai Zoo Shanghai China; 3 Jiading Agriculture Technology Extension Service Center, Jiading District, Shanghai 201403, China Jiading Agriculture Technology Extension Service Center Shanghai China

**Keywords:** Ant-like stone beetles, East Asia, *Euconnus*, identification key, new record, new synonym, new taxa, Stenichnini, taxonomy, urban ecosystems

## Abstract

The subfamily Scydmaeninae (Coleoptera: Staphylinidae) is reported from Shanghai, China for the first time. Three species of the genus *Euconnus* Thomson were recognized: *E.* (*s. str.*) *dulcis* Sharp, 1886, *E.(s. str.)imparitus***sp. nov.** (type locality: Waigang Town, Jiading District), and *E.(s. str.)magnoculus***sp. nov.** (type locality: Shanghai Zoo, Hongqiao District). The new species are described, and diagnoses and illustrations of the habitus and important diagnostic features for all taxa are provided for ready identification. Furthermore, *Euconnuscerastiventris* Vit, 2006, **syn. nov.** is placed as a junior synonym of *E.dulcis*. A key to *Euconnus* species that occur in Shanghai is provided.

## ﻿Introduction

The potential role of Shanghai, a mega-international metropolis in China, as a habitat for undescribed insect species has been consistently demonstrated over the past decade. A number of novel species, predominantly from the order Coleoptera (beetles), have been documented (Wang et al. 2017; Yin et al. 2017; Song et al. 2018, 2019). A continued survey of the rove beetle fauna in Shanghai, conducted primarily by a group of researchers affiliated with Shanghai Normal University, has resulted in the discovery of a small number of specimens belonging to the subfamily Scydmaeninae (Coleoptera: Staphylinidae), a large taxonomic group previously unreported in the city (O’Keefe and Li J-K 1998; Schülke and Smetana 2015). This study focuses on the genus *Euconnus* Thomson and reports findings on three species. The first two species, each represented by a single male specimen collected from Jiading and Hongqiao districts, respectively, are described herein as new to science. The third one, represented by both sexes collected from Minhang and Songjiang districts, has been identified as conspecific with a previously known species that exhibits a broad distributional range across East Asia. Although at least two additional potentially distinct species were observed, these are represented solely by females and are not presented in detail. This paper marks the first scientific record of the subfamily Scydmaeninae in Shanghai and highlights the significance of fragmented habitats for preserving previously undocumented biodiversity within urban ecosystems.

## ﻿Material and methods

All specimens examined in this study are housed in the Insect Collection of Shanghai Normal University, Shanghai, China (SNUC). The label data for these specimens are quoted verbatim. Dissected parts were mounted in Euparal on plastic slides pinned with the specimen. Habitus images of the beetles were captured using a Canon EOS R5 camera equipped with a 10 × Mitutoyo M Plan Apo lens, with three 10W LED bulbs (5500 K) serving as the light source. Images of morphological details were taken using a Canon G9 camera mounted on an Olympus CX31 microscope under reflected or transmitted light. Image stacking was performed using Helicon Focus v. 8.2.0 Pro, and all images were edited and compiled into plates using Adobe Photoshop CC 2020.

Measurements were conducted as follows: head length was measured from the anterior margin of the clypeus to the head base, excluding the cervical constriction; head width was measured across the eyes; pronotum length was measured along the midline, and pronotum width was measured at its maximum width; elytra length was measured along the suture, while elytra width was measured at its maximum width across both elytra; total body length was measured from the apex of clypeus to the apex of elytra. In descriptions, paired appendages are treated as singular. Following Chandler (2001) and Yin (2022), the abdominal segments are numbered in Arabic (starting from the first visible segment) and Roman (reflecting true morphological position) numerals, e.g., sternite 1 (III).

## ﻿Taxonomy

### ﻿Family Staphylinidae Latreille, 1802

**Subfamily Scydmaeninae Leach**, **1815**

**Supertribe Scydmaenitae Leach**, **1815**

**Tribe Stenichnini Fauvel, 1885**

**Genus *Euconnus* Thomson, 1859**

#### ﻿ Euconnus (s. str.) dulcis

Taxon classificationAnimaliaColeopteraStaphylinidae

 Sharp, 1886

E881AB71-06EF-53F5-89CB-6436ABF171BD

Figs 1 , 4

Euconnus dulcis Sharp, 1886: 47; Hoshina 2019: 201. Type locality: Nagasaki.
Euconnus (*s. str.*) dulcis Sharp; Jałoszyński 2022: 3; Byeon et al. 2023: 321.
Euconnus chinensis Li, J.-K. & Wang, Z.-Y., 1993: 163 (nec. E.chinensis Franz, 1985: 114). Type locality: Ningguo. Syn. nov.
Euconnus cerastiventris Vit, 2006: 75 (replacement name for E.chinensis Li, J.-K. & Wang, Z.-Y.). Syn. nov.

##### Material examined

**(5 exx.).** • 1 ♂: ‘China: Shanghai City, Minhang Dist., 31°01'N, 121°28'E, alt. 4 m, 6.iv.2014, Xiao-Bin Song leg.’; • 2 ♀♀: ‘China: Shanghai City, Songjiang Dist., East Sheshan, 9.iv.2021, Xiao-Bin Song leg.’; • 3 ♀♀: ‘China: Shanghai City, Jiading Dist., Liudao, 31°29'38"N, 121°14'3"E, alt. 3 m, 3.x.2023, Yin & Zhou leg.’. (all in SNUC).

##### Diagnosis.

**Figure 1.**
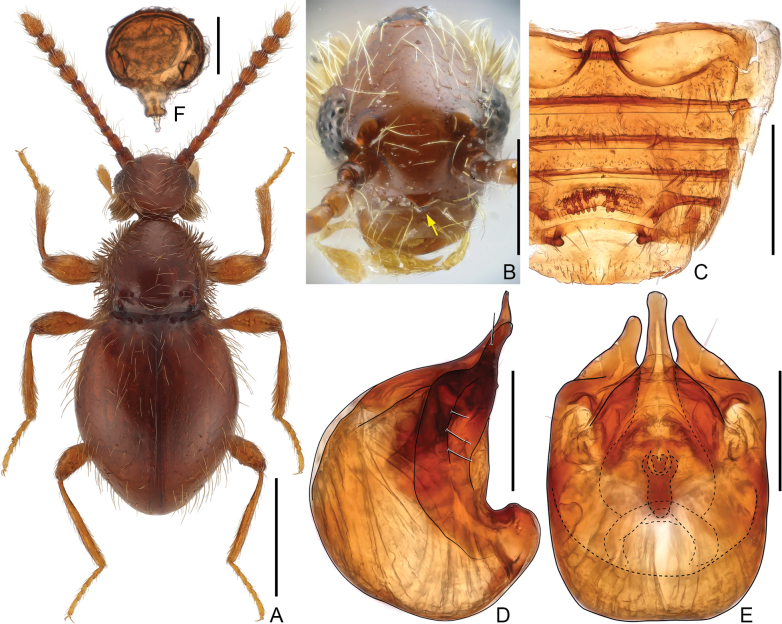
*Euconnus* (*s. str.*) *dulcis* (**B–E** male **A, F** female) **A** dorsal habitus **B** head, in anterior view, showing clypeal angulation **C** sternites, showing modification on 4 (VI) and 5 (VII) **D, E** aedeagus, lateral (**D**) and ventral (**E**) **F** spermatheca, lateral. Scale bars: 0.5 mm (**A**); 0.2 mm (**B, C**); 0.1 mm (**D, E**); 0.05 mm (**F**).

**Male.** Body length 1.4–1.7 mm. Dorsum of body finely punctate. Thick bristles present on tempora and sides of pronotum, especially dense on tempora. Anterior margin of clypeus (Fig. 1B) angularly prominent at middle. Antenna elongate, club loosely formed by apical four moderately enlarged antennomeres, occupying about half of antennal length. Pronotum bell-shaped, with two asetose basolateral pits connected by shallow transverse impression. Each elytron with two closely-placed basal pits. Abdomen greatly modified (Fig. 1C), sternite 4 (VI) and 5 (VII) each with two posterolateral nodules directed posteromedially, area between nodules on sternite 4 filled with peg-like granules distributed roughly in two transverse rows. Aedeagus (Fig. 1D, E) with apical projection much longer than parameres; endophallus armature composed of group of symmetric sclerites; parameres each greatly broadened, bearing two long setae near apex and three similar setae along lateral margin. **Female** (Fig. 1A). External morphology similar to male. Abdomen unmodified. Spermatheca (Fig. 1F) spherical; spermathecal duct broadened at base.

##### Description.

See Vit (2006) and Jałoszyński (2022). Measurement for Shanghai population: male body length 1.48 mm; length/width of head 0.35 mm/0.34 mm, pronotum 0.38 mm/0.37 mm, elytra 0.78 mm/0.63 mm, length of antenna 0.81 mm, club 0.42 mm, length of aedeagus 0.27 mm; female body length 1.49–1.53 mm; length/width of head 0.37–0.39 mm/0.34–0.38 mm, pronotum 0.41–0.43 mm/0.39–0.41 mm, elytra 0.86–0.88 mm/0.67–0.71 mm, length of antenna 0.77–0.80 mm, club 0.36–0.38 mm, maximum diameter of spermatheca 0.27 mm.

##### Distribution.

East China: Anhui, Shanghai (Minhang, Songjiang, Jiading) (Fig. 4A); Japan: Honshu, Kyushu; South Korea: Jeju. New record for Shanghai.

##### Biology.

Adult specimens were obtained by sifting grass roots, and mixed bush and bamboo leaf litter (Fig. 4B–D).

##### Remarks.

This species exhibits a wide distribution across East Asia and is readily distinguishable by an angulate clypeus in both sexes, a sexually dimorphic abdomen in the male, and a distinctive morphology of the aedeagus. The descriptions and illustrations presented by Vit (2006) and Jałoszyński (2022) offer compelling evidence supporting the proposed synonymy. The spermatheca (Fig. 1F) of this species is illustrated for the first time.

#### ﻿ Euconnus (s. str.) imparitus

Taxon classificationAnimaliaColeopteraStaphylinidae

 Zi-Wei Yin, Ting Feng & De-Yao Zhou sp. nov.

B9BF696E-0AF6-5B24-B48D-6068CC860011

https://zoobank.org/E0AF44E1-5C56-4F4A-996E-DCD68A6448AB

Figs 2 , 4

##### Type material

**(1 ex.). *Holotype*: China**: • ♂: ‘China: Shanghai, Jiading, Waigang To., Quanjing Vill., 31°22'29"N, 121°8'22"E, late vii.2018, light trap, D-Y Zhou leg., 嘉定外冈泉泾村测报灯周德尧’ (SNUC).

##### Diagnosis.

**Male.** Habitus elongate; body length approximately 1.7 mm. Head and elytra finely punctate, subglabrous, with sparse long setae; punctation and setae of pronotal disc similar to those of head and elytra, lateral margins densely setose and with numerous thick bristles. Head subspherical, eyes anteriorly situated, tempora much longer than eyes. Antennae elongate, antennomeres elongate, clubs loosely formed by apical four enlarged antennomeres. Pronotum lacking antebasal pits, transverse impression, or sublateral carinae; broadest slightly posterior to middle. Tarsomere 1 of protarsus modified, ventrally protruding to form apically truncate projection. Aedeagus moderately elongate, dorso-ventrally symmetric; compressor plate in ventral view with two lateral lobes; apical projection of median lobe broad at base and narrowing apically; parameres broadened before apices, each paramere with three macrosetae at apex. **Female.** Unknown.

##### Description.

**Figure 2.**
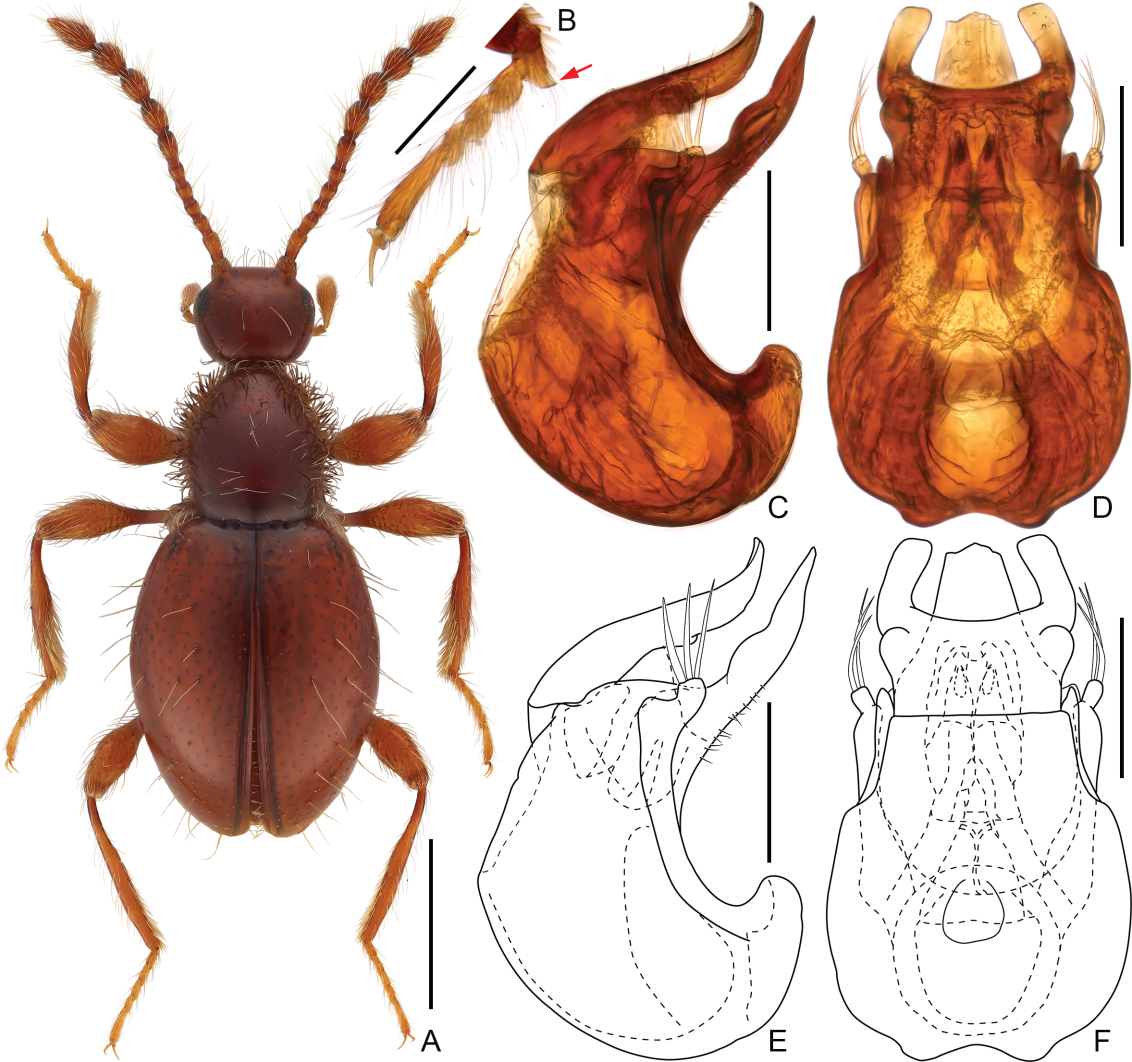
*Euconnus(s. str.)imparitus* sp. nov., male **A** dorsal habitus **B** protarsus **C–F** aedeagus, lateral (**C, E**) and ventral (**D, F**). Scale bars: 0.5 mm in (**A**); 0.1 mm (**B–F**).

**Male.** Body (Fig. 2A) length 1.66 mm; body uniformly reddish-brown, mouthparts and tarsi paler in color. Setae long and suberect, sparse on head and pronotal and elytral discs, sides of pronotum with dense, thick bristles. Dorsum of body finely and sparsely punctate, almost glabrous.

Head subrounded, as long as wide, broadest at eyes, length and width 0.36 mm; vertex and frons confluent, weakly convex; supraantennal tubercles barely prominent; eyes relatively small, barely convex and finely faceted. Punctures on vertex and frons fine; setae long and sparse, suberect, tempora much longer than eyes, lacking bristles. Antenna elongate, length 0.93 mm, club 0.48 mm; antennomeres 1–7 each elongate, 2 and 7 longest, 8–11 broader than 2–7, enlarged, form loose club.

Pronotum in dorsal view slightly longer than wide, subglobose, broadest slightly posterior to middle and narrowing anteriorly and posteriorly, length 0.4 width 0.41 mm; lacking lateral antebasal pits, transverse antebasal groove and sublateral carinae. Punctures on pronotal disc rather fine, almost glabrous; setae sparse and long, laterally obscured by dense, long and thick bristles.

Elytra suboval and slightly flattened, broadest approximately at middle, length 0.94 mm, width 0.73 mm, length/width 1.29; basal impressions shallow, with four small, asetose basal pits, humeral calli weak; apices of elytra separately rounded. Punctures on elytral disc fine and shallow; sparse setae long and suberect. Metathoracic wings fully developed, functional.

Meso- and metaventrite fused. Mesoventral intercoxal process posteriorly extending far beyond level of posterior margin of mesocoxae. Metaventral intercoxal process broad, emarginate at middle.

Legs long and slender; protarsus with tarsomere 1 projecting ventrally, forming short, apically truncate lobe (Fig. 2B).

Aedeagus (Fig. 2C–F) moderately elongate, dorso-ventrally almost symmetric, length 0.34 mm, in ventral view median lobe with long, apically narrowing projection greatly curved dorsally, with two rows of fine setae along dorsal wall of projection; compressor plate broadened in dorso-ventral view, with pair of short and broad lateral lobes, plate inclined to apically-projected median lobe in lateral view and curved dorsally; endophallus armature composed of pairs of elongate and sclerotized plates and membranous structures; parameres elongate, narrow, extending just beyond base of apical projection of median lobe, areas before apices greatly broadened and then abruptly narrowing apically, each paramere with three long macrosetae at apex.

**Female.** Unknown.

##### Comparative notes.

This species is closely related to *Euconnusimpar* Sharp, distributed in Japan and South Korea, due to similar morphological features (elongate habitus, subglabrous head and elytra, dense bristles on pronotum sides, loosely assembled tetramerous antennal clubs), and particularly the modified male protarsi. However, *E.imparitus* is clearly distinguishable by its significantly different aedeagal structure. The aedeagus of the new species comprises a compressor plate with two short, blunt lateral lobes (vs. with two long, rod-like lobes in *E.impar*), a dorso-apical projection of the median lobe that narrows apically in ventral view (vs. apical projection broad and blunt in *E.impar*), and parameres broadening near the apices, each bearing three apical macrosetae (vs. parameres slender throughout, each with two long apical setae in *E.impar*). Additionally, the apical four antennomeres forming the club of this species appear relatively more elongate than those of *E.impar*.

##### Distribution.

East China: Shanghai (Jiading) (Fig. 4A).

##### Biology.

The male was taken from a mixed light trap sample deployed in an agricultural setting (Fig. 4E).

##### Etymology.

The specific epithet is derived from a combination of *E.impar*, a closely related species, and the Latin suffix “-*itus* (-*a*, -*um*)”, denoting an affinity between these two species.

#### ﻿ Euconnus (s. str.) magnoculus

Taxon classificationAnimaliaColeopteraStaphylinidae

 Zi-Wei Yin, Ting Feng & De-Yao Zhou sp. nov.

72F9430C-5400-54B7-A616-51F1AA31E520

https://zoobank.org/5A02E349-93CC-44C7-97F3-A3012053D692

Figs 3 , 4

##### Type material

**(1 ex.). Holotype: China**: • ♂: ‘China: Shanghai, Hongqiao Dist., Shanghai Zoo, 31.198056°N, 121.354964°E, alt. 10 m, 07.vi.2023, Ting Feng leg., 上海动物园封婷采’ (SNUC).

##### Diagnosis.

**Male.** Body length approximately 1.5 mm. Eyes greatly prominent, approximately 1.4 × as long as tempora. Terminal four antennomeres greatly enlarged and forming distinct club, occupying approximately 5.5/10 of antennal length. Sides of elytra distinctly narrowing posteriorly from broadest point. Aedeagus with compressor plate elongate and subfusiform in ventral view; apical projection of median lobe rounded and greatly protruding in ventral view, curved dorsally in apical portion in lateral view; median lobe with pair of apically rounded lateral projections, and transversely rhomboidal plate on ventral wall; broad and elongate parameres narrowing from bases toward apices, each with two long setae at apex and three similar long setae along apical 2/5. **Female.** Unknown.

##### Description.

**Figure 3.**
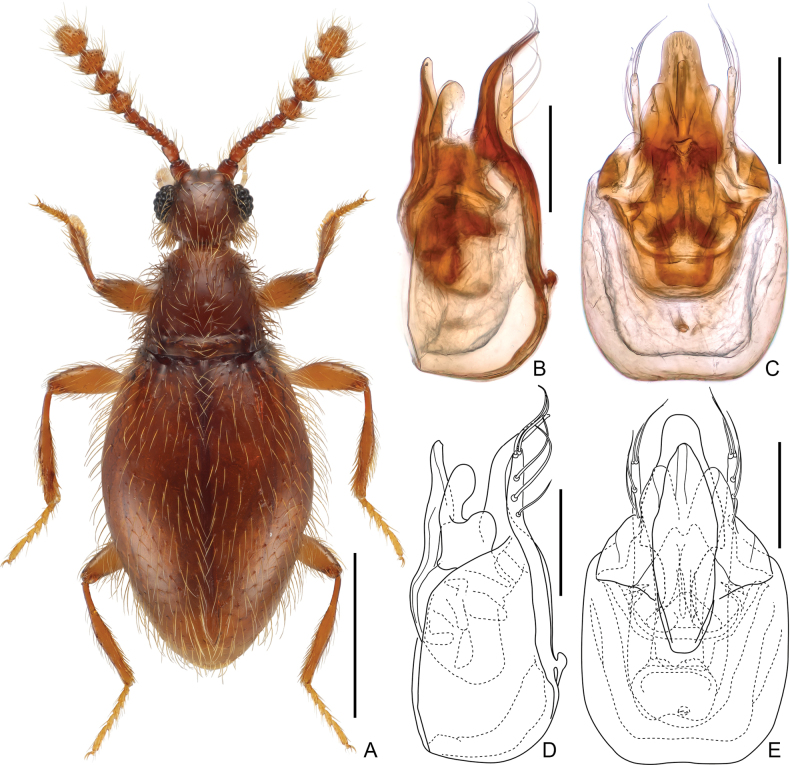
*Euconnus(s. str.)magnoculus* sp. nov., male **A** dorsal habitus **B–E** aedeagus, lateral (**B, D**) and ventral (**C, E**). Scale bars: 0.5 mm (**A**); 0.1 mm (**B–E**).

**Male.** Body (Fig. 3A) length 1.52 mm; body uniformly reddish-brown, mouthparts and tarsi paler in color. Setae long and suberect, tempora of head and sides of pronotum with dense, thick bristles. Dorsum of body finely and sparsely punctate.

Head roundly rhomboidal, as long as wide, broadest at eyes, length and width 0.31 mm; vertex and frons confluent, weakly convex; supraantennal tubercles barely prominent; eyes large, strongly convex and coarsely faceted. Punctures on vertex and frons fine; setae long and sparse, suberect, additionally tempora with long bristles directed posteriorly. Antenna moderately short, length 0.56 mm, club 0.31 mm; antennomeres 1 and 2 subcylindrical, elongate, 3–7 compact, gradually larger, 8–11 greatly enlarged, conical, 11 largest, distinctly shorter than 9 and 10 combined.

Pronotum in dorsal view subtrapezoidal, broadest at base and strongly narrowing anteriorly, length 0.35 mm, width 0.38 mm; lateral antebasal pits small but distinct, asetose, connected by transverse antebasal groove. Punctures on pronotal disc fine; setae long, obscured by dense, long and thick bristles especially on sides.

Elytra suboval and slightly flattened, broadest approximately at middle, length 0.89 mm, width 0.63 mm, length/width 1.39; basal impressions shallow but distinct, with four small, asetose basal pits, humeral calli elongate; apices of elytra separately rounded. Punctures on elytral disc fine and shallow; setae long, sparse and suberect. Metathoracic wings fully developed, functional.

Meso- and metaventrite fused. Sides of mesoventral intercoxal process posteriorly divergent, form pair of ridges, similar to condition in *E.maklinii* (Mannerheim) (Jałoszyński 2021: fig. 8). Metaventral intercoxal process relatively narrow.

Legs long and slender; unmodified.

Aedeagus (Fig. 3B–E) moderately elongate, dorso-ventrally almost symmetric, length 0.31 mm, in ventral view median lobe with abruptly delimited and long, broad apical projection greatly curved dorsally, rounded at apex; compressor plate relatively narrow and subfusiform in dorso-ventral view, with narrowed anterior and posterior margins, connected in parallel to median lobe in lateral view; lateral projections broad and partially sclerotized, curved dorsally, with round apices; endophallus armature composed of pairs of complex, symmetric sclerotized plates and large, transversely rhomboidal plate, its apical margin with two admesal roundly acute projections; parameres broad and elongate, narrowing from bases toward apices, each with two long setae at apex, and three similar long setae along apical 2/5.

**Female.** Unknown.

##### Comparative notes.

Among the East Asian *Euconnus* fauna, several species exhibit a similar general shape of the aedeagus. These include *E.efferus* Franz from China (Taiwan) (Franz 1985), *E.deprecator* Kurbatov from the Russian Far East (Kurbatov 1993), and *E.akane* Hoshina from Japan (Hoshina 2020). Despite the similarities, these species are distinctly differentiated by their endophallus armature, which comprises asymmetric sclerites. In contrast, the new species possesses an aedeagus that is almost symmetric both externally and internally. Additionally, similarly symmetric aedeagi are found in *E.kelantanensis* Franz from West Malaysia and *E.parakelantanensis* Franz from north-central Thailand; however, these species are considerably smaller, measuring only 1.20 mm and 1.10 mm in length, respectively (Franz 1970, 1985).

##### Distribution.

**Figure 4.**
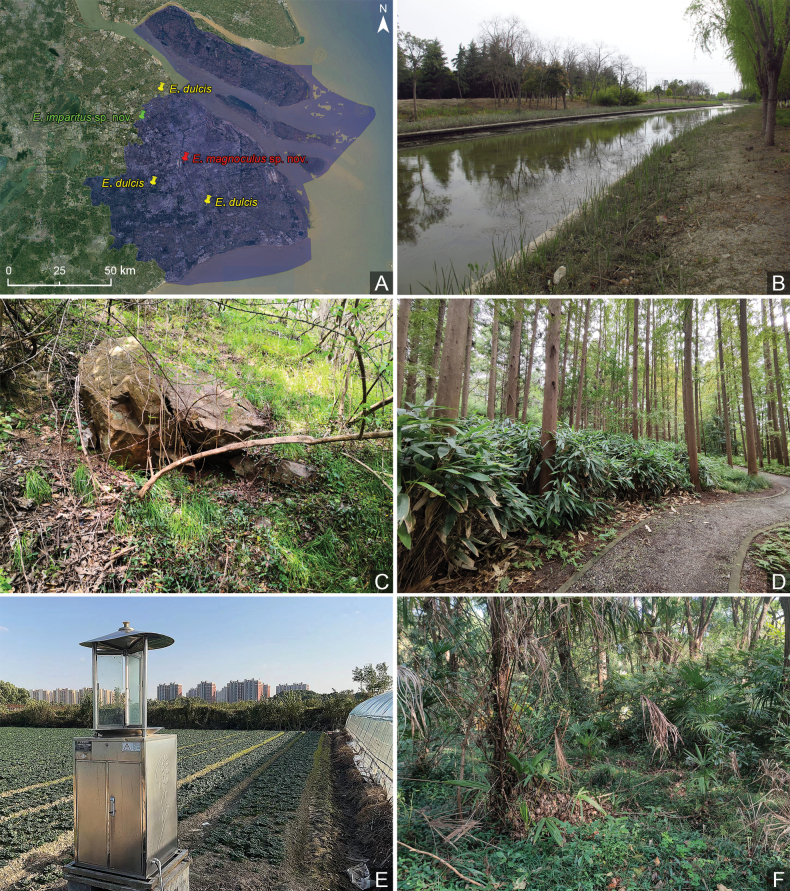
Distribution and collecting circumstances of *Euconnus* species in Shanghai **A** distribution of the three species **B–D** collecting circumstance of *E.dulcis* at Minhang (**B**), Sheshan (**C**), and Liudao (**D**), *E.imparitus* sp. nov. at Quanjing Village (**E**), and *E.magnoculus* sp. nov. at Shanghai Zoo (**F**).

East China: Shanghai (Hongqiao) (Fig. 4A).

##### Biology.

The specimen was collected from a leaf litter sample taken in a secondary mixed forest within Shanghai Zoo (Fig. 4F).

##### Etymology.

The name is a combination of the Latin adjective “*magnus* (great, large)” and noun “*oculus* (eye)”, referring to the large eyes of this species.

### ﻿Key to *Euconnus* species occurring in Shanghai (male)

**Table:** 

1	Head lacking thick bristles on tempora (Fig. 2A); antennal clubs comprising elongate antennomeres (Fig. 2A); male protarsomere 1 modified, ventrally expanded to form blunt projection (Fig. 2A, B)	***E.imparitus* sp. nov.**
–	Head with thick bristles on tempora; antennal clubs formed by cornicle or submoniliform antennomeres; male protarsomere 1 simple, lacking modifications	**2**
2	Eyes greatly convex, much longer than tempora (Fig. 3A); antennomeres 8–10 conical (Fig. 3A); anterior margin of clypeus smooth, lacking tubercle at middle; sternites 4 (VI) and 5 (VII) simple, lacking modifications	***E.magnoculus* sp. nov.**
–	Eyes moderately convex, distinctly shorter than tempora (Fig. 1A); antennomeres 8–10 submoniliform; anterior margin of clypeus with angulate tubercle at middle (Fig. 1B); sternites 4 (VI) and 5 (VII) modified, each with pair of lateral tubercles on posterior margin (Fig. 1C)	***E* . *dulcis* Sharp**

## Supplementary Material

XML Treatment for Euconnus (s. str.) dulcis

XML Treatment for Euconnus (s. str.) imparitus

XML Treatment for Euconnus (s. str.) magnoculus

## ﻿Additional information

### Author ORCIDs

Zi-Wei Yin https://orcid.org/0000-0001-6659-9448

Ting Feng https://orcid.org/0009-0007-0869-6654

De-Yao Zhou https://orcid.org/0000-0002-2014-9967

### Data availability

All of the data that support the findings of this study are available in the main text.
